# Direct arterial puncture for hemodialysis, a neglected but simple and valuable vascular access

**DOI:** 10.1186/s12882-022-02836-1

**Published:** 2022-06-23

**Authors:** Chun-Yan Sun, Mi Zhong, Li Song, Ying-Gui Chen, Zi-lin Quan, Li-Yan Zhao, Dong-Mei Cui, Xia Fu

**Affiliations:** 1 Division of Nephrology, Guangdong Provincial People’s Hospital, Guangdong Academy of Medical Sciences, Guangzhou, 510080 China; 2 School of Nursing, Southern Medical University, Guangzhou, 510515 China; 3 The Eighth Affiliated Hospital, Sun Yat-Sen University, Shenzhen, 518033 China

**Keywords:** Hemodialysis, Vascular access, Direct arterial puncture, Effect

## Abstract

**Introduction:**

The purpose of this study is to present the prevalence and effects of direct arterial puncture (DAP) for hemodialysis patients, and to introduce optimal option for the vascular access (VA) in certain hemodialysis patients with poor condition of vascular or cardiac function in a compelling situation.

**Methods:**

This was a cross-sectional study. Demographic characteristics and laboratory data were extracted from the health care system. Relevant DAP information was collected by a questionnaire. Case-control matching was performed to compare the hemodialysis adequacy between DAP and other VAs.

**Results:**

A total of 526 patients were selected for analysis by convenience sampling, of which 38 patients relied https://www.baidu.com/link?url=eaDh8Hn-yZGJyDB0_h4zBenKd7qY1yX-KNxO-qU49gktQOGTJJg3slTjIbG095st4hRfprQIHRjfhfeGOZyH73y8tvSUCwMmvWbUhyix2ZKon DAP for hemodialysis. The main reasons using DAP for hemodialysis included the cost of arteriovenous access creation or maintenance in 19(50%) patients and the poor condition of vascular or cardiac function in 14 (39.5%) patients. Some complications of DAP occurred, such as aneurysm or pseudoaneurysm in 16(42.1%) patients, infiltration in 12 (31.6%) patients. Differences in hemodialysis adequacy were not statistically significant between DAP and other types of VA.

**Conclusion:**

In conclusion, DAP can meet the need of prescription hemodialysis, yet it has several limitations. Although the patients in our study were long-term dependent on DAP for hemodialysis with various reasons, we do not recommend DAP as a long-term vascular access if better options are available. However, DAP should not be overlooked to be a supplemental VA for hemodialysis with adequate blood flow and availability for individuals with poor condition of vascular or cardiac function in a compelling situation.

**Supplementary Information:**

The online version contains supplementary material available at 10.1186/s12882-022-02836-1.

## Introduction

The number of maintenance hemodialysis (MHD) patients worldwide is increasing rapidly each year [1]. Well-functioning vascular access (VA) is the key to ensure sufficient hemodialysis and to improve the prognosis of MHD patients [2]. There are three acknowledged types of hemodialysis VA: arteriovenous fistula (AVF), arteriovenous graft (AVG) and central venous catheter (CVC), and AVF has been recommended as "fistula first" by lots guidelines for fewer complications and a long lifespan [3]. Life plan for the choice of VA types was recommended by the updated guidelines rather than the previous “fistula first” [4]. The right one is the best, when it comes to the optimal VA for hemodialysis.

**Fig. 1:**
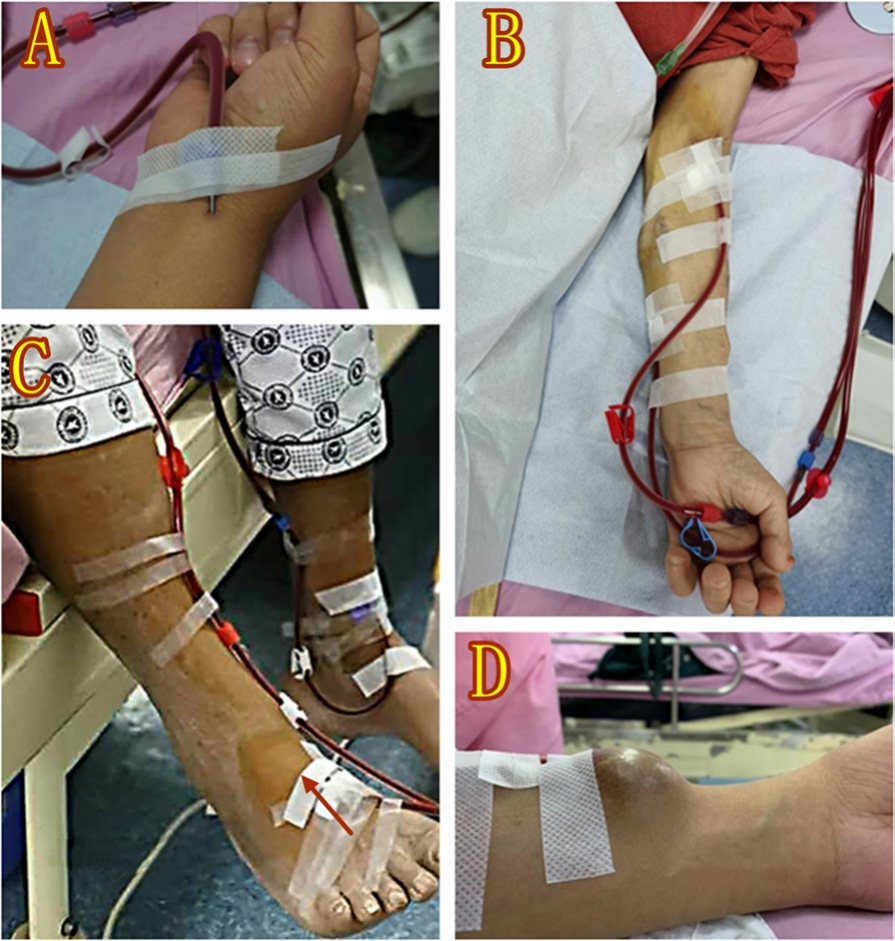
Different sites of direct arterial puncture. **A** Radial artery; **B** Brachial artery; **C** dorsalis pedis artery; **D** Arteriovenous anastomosis

Because of the rapid growth of the aging population and the high prevalence of comorbidity, particularly diabetes mellitus, it’s really hard for patients undergoing MHD to create and maintain a well-functional AVF [5]. Some patients exhaust their peripheral veins and do not retain the venous capital necessary for fistula creation [6], and some other with poor cardiac function cannot afford the burden of arteriovenous shunt on the heart, even if their peripheral veins and/or arteries were available [7]. Since a renal transplant is not readily available, patients virtually face death in the absence of dialysis therapy [8]. Hence, it is critically important that VA is available to successfully receive the hemodialysis therapy. In China, direct arterial puncture (DAP) is chosen for 2-needles hemodialysis in some hemodialysis units to deal with the cases where arteriovenous access or catheter cannot be established (Fig. 1). DAP is used for the arterial line and peripheral veins or catheter is used as the venous line to return blood. DAP is often performed with different gauge metal needles or plastic cannulas depending on patients’ artirial condition. In case of a plastic cannula, the core steel needle will be removed after the plastic sleeve is introduced into the artery. The plastic cannula can be introduced into the artery according to the depth and diameter of vessel measured by ultrasound, which is unnecessary while patient's arteries are superficial.

Arterial puncture is a commonly performed invasive procedure and allows blood pressure measurement, blood sampling for blood gas analysis, and can be used for guiding fluid therapy in critically ill or surgical patients [9]. It also can be adopted for hemodialysis by appropriately sized and material needles, which accounted for 0.1% of VAs in Japan [10]. Similar to the practice of DAP is cannulation of a superficialized artery which accounted for 1.8% of VAs in Japan and was reported to be a simple and safe technique with acceptable durability and complication rate [11]. However, it isn’t simple and practicable like DAP because it is not worked until 3 weeks after surgery. This article introduces the prevalence and relevant information about DAP and potential effects of DAP, and aims to introduce the one more option for hemodialysis VA in certain patients.

## Method

### Study design and participants

This cross-sectional study was carried out from September to November of 2020 in blood purification centers in mainland China. The criteria for participants to be eligible for the study were as follows: (1) DAP technique was used for hemodialysis; (2) 18 years or older; (3) without any psychiatric diagnoses based on the SCID-5. Participants were excluded for key missing data.

### Data Collection and Instrument

Demographic characteristics and most recent laboratory data was extracted from the health care system. Relevant DAP information were collected by a questionnaire. Charlson Comorbidity Index (CCI) was evaluated by a nephrologist with more than 5-year work experience. Information concerning DAP was evaluated by self-designed questionnaire consisting of duration, reasons, location, time of achieving hemostasis after needle withdrawal, complications, Numeric Rating Scales (NRS) for pain. The NRS [12] is a 11-point scale where the starting point is an extreme of no pain and the end point is the worst pain. A corresponding number was selected on the basis of the pain degrees, which were divided into mild pain (1-3), moderate pain (4-6) and severe pain (7-10). The NRS is superior in this respect for it has greater sensitivity to change and is the most popular for patients when asked to quantify their pain degrees. Urea reduction ratio (URR)= (predialysis blood urine nitrogen-postdialysis blood urine nitrogen)/predialysis blood urine nitrogen. single-pool Kt/V-urea (spKt/V)= -ln(R-0.008×T)+4-3.5R)×0.55×Weight loss/V. R is the ratio of postdialysis to predialysis blood urine nitrogen; V is body water volume and Weight loss is expressed in the same units; and T is hemodialysis time in hours [13].

This project was authorized by the Ethics Committees of Guangdong Provincial People's Hospital (No. GDREC2019652H(R1)), and the study was conducted in full compliance with national ethical guidelines. All patients signed informed consent.

### Statistical methods

Continuous variables are expressed as the mean+1 standard deviation (SD) or median (interquartile range, IQR) as appropriate, t test, ANOVA or Mann-Whitney U test were used to judging the difference between the two groups of data. Discrete variables are presented as percentages. Differences between groups were determined by Chi-square test or Fisher exact test. Case-control matching methods were performed to select control cases from patients with AVF, AVG and CVC respectively to compare the hemodialysis adequacy with patients adopting DAP technique. *P*-values are two sided, with *P*<0.05 considered to indicate a statistically significant difference. IBM SPSS software (version 26.0, Chicago, IL) was used to perform the analysis.

## Results

### Characteristics of study participants

A total of 526 patients were selected for analysis by convenience sampling, of which 38 patients relied on a vascular access of DAP for hemodialysis. In the 38 patients, 29 patients were males and 9 patients were women; the average age was 58.95±11.86 years; the comorbidities were chronic glomerulonephritis in 15(39.5%) patients and diabetic nephropathies in 8 patients (21.1%). Significant differences between the four groups were observed regarding gender, age, primary disease, CCI, blood pump flow rate and albumin hemoglobin. Detailed data were shown in Table 1.

**Table 1:** Demographics characteristics of participants

Items	DAP (*n*=38)	AVF (*n*=374)	AVG (*n*=33)	CVC (*n*=81)	*P*
Gender, n (%)					0.009
Male	29(76.3)	220(58.8)	16(48.5)	37(45.7)	
Female	9(23.7)	154(41.2)	17(51.5)	44(54.3)	
Age, year, mean±SD	58.95±11.86	58.66±14.34	66.39±13.57	62.44±17.25	0.009
BMI, mean±SD	21.20±3.76	21.77±3.99	21.40±3.15	20.83±2.95	0.204
Primary disease, n(%)					<0.001
Chronic glomerulonephritis	15(39.5)	126(33.7)	7(21.2)	8(9.9)	
Diabetic nephropathy	8(21.1)	99(26.5)	11(33.3)	43(53.1)	
Hypertensive nephropathy	5(13.2)	70(18.7)	7(21.2)	12(14.8)	
Obstructive nephropathy	5(13.2)	61(16.3)	8(24.2)	8(9.9)	
Others	5(13.2)	18(4.8)	0(0.0)	10(12.3)	
CCI, median (IQR)	3(2,5)	3(2,3)	3(2,4)	3(2,4)	0.012
Blood pump flow speed, ml/min, mean±SD	224.21±26.57	217.83±19.09	212.12±15.36	200.25±10.24	<0.001
ALB, g/L, mean±SD	35.96±4.42	36.86±2.80	36.37±2.18	35.83±2.71	0.014
Hemoglobin, g/L, mean±SD	108.24±15.75	108.12±12.39	107.27±10.86	108.06±12.33	0.986
WBC,10^9/L, mean±SD	5.94±2.18	6.16±2.31	6.48±2.31	6.19±2.70	0.812
PLT,10^9/L, mean±SD	171.66±68.48	175.99±49.11	173.06±55.07	175.23±42.69	0.954
CRP, mg/L, median (IQR)	5.3(1.00, 12.50)	3.27(1.40,7.10)	2.66(0.88,8.22)	5.90(2.00,8.00)	0.095
Calcium, mmol/L, mean±SD	2.26±0.18	2.30±0.20	2.30±0.16	2.27±0.19	0.567
Potassium, μmol/L, mean±SD	4.68±0.63	4.76±0.71	4.81±0.64	4.66±0.69	0.589
Phosphate, μmol/L, mean±SD	1.84±0.53	1.90±0.57	1.82±0.45	2.00±0.57	0.306
iPTH, pg/ml, median (IQR)	241.60(135.70, 355.90)	312.50(168.35,538.85)	335.5(198.80,618.10)	286.50(141.10,426.50)	0.572
UA, μmol/L, mean±SD	440.22±112.15	462.93±99.74	437.47±78.36	448.85±123.20	0.359
LDL,mmol/L, mean±SD	2.80±1.43	2.63±0.87	2.40±0.76	2.52±0.87	0.228

*DAP* Direct arterial puncture; *AVF* Arteriovenous Fistula; *AVG* Arteriovenous Graft; *CVC* Central Venous Catheter; *SD* Standard deviation; *BMI* Body mass index; *IQR* Interquartile range; *CCI* Charlson Comorbidity Index; *ALB* Albumin Hemoglobin; *WBC* White blood cell; *PLT* Platelet count; *CRP* C-reactive protein; *iPTH* Intact parathyroid hormone; *UA* Uric acid; *LDL* Low-density lipoprotein

### Relevant DAP information

All DAP procedures were conducted by senior nurses. Among the 38 patients, The cannulation site on radial artery was for 24 patients (63.2%). The cannulation site on brachial artery was for 7(18.4%). About the reasons receiving hemodialysis by DAP, 19(50%) patients claimed to be unable to afford the cost of AVF creation or maintenance. 15(39.5%) patients expressed the reason for receiving DAP was that the matured arteriovenous fistula cannot be created because of the poor condition of cardiac function. The manual time-to-hemostasis compression required 0.5-1 h in 16 patients (42.1%). There were aneurysm or pseudoaneurysm complications in 16 patients (42.1%). The NRS evaluation was mainly mild pain in 29 patients (76.3%). The average level of URR was 0.65±0.12, and the average spKt/V was 1.33±0.30. For details, see Table 2

**Table 2:** Relevant DAP information in patients undergoing hemodialysis

Items	All(*n*=38)
DAP for permanent hemodialysis access, n (%)	35(92.1)
DAP duration, days	1428(141, 2950)
Location of DAP, n (%)	
Radial artery	24**(**63.2**)**
Brachial artery	7**(**18.4**)**
Arteriovenous anastomosis	6**(**15.8**)**
Femoral artery	1**(**2.6**)**
Previous vascular aceess before DAP, Minimum~Maximum	2~7
Reasons, n (%)	
Consideration for the cost and of AVF creation or maintenance	19**(**50**)**
Poor condition of cardiac function or vascular	15**(**39.5**)**
Others	6**(**15.8**)**
The time-to-hemostasis compression after needle withdrawal, n (%)	
0.5-1h	16**(**42.1**)**
1-2h	5**(**13.2**)**
2-4h	9**(**23.7**)**
4-6h	8**(**21.1**)**
Complications, n (%)	25(65.8)
Infiltration	12(31.6)
Hematoma	3(7.9)
Aneurysm or pseudoaneurysm	16(42.1)
Arteriosclerosis	1(2.6)
Infection	3(7.9)
NRS score, n (%)	
1-3	29(76.3)
4-6	9(23.7)

*DAP* Direct arterial puncture; *AVF* Arteriovenous Fistula; *NRS* Numeric Rating Scales

### Comparison of hemodialysis adequacy in patients with different types of vascular access

The patients with DAP were matched with patients with AVF, AVG and CVC respectively in the corresponding hemodialysis center. The details before and after matching were shown in the supplementary tables, and the adequacy of hemodialysis after matching were shown in the Table 3.

**Table 3:** Comparison of hemodialysis adequacy after case-control matching

Vascular access	SpKt/V	URR
DAP VS AVF		
DAP(n=38)	1.36±0.30	0.66±0.12
AVF(n=38)	1.47±0.35	0.68±0.08
*P*	0.131	0.250
DAP VS AVG		
DAP(n=22)	1.41±0.29	0.67±0.09
AVG(n=22)	1.43±0.42	0.69±0.07
*P*	0.859	0.474
DAP VS CVC		
DAP (n=22)	1.41±0.28	0.67±0.13
CVC (n=22)	1.24±0.37	0.69±0.06
*P*	0.072	0.468

*DAP* Direct arterial puncture; *AVF* Arteriovenous Fistula; *AVG* Arteriovenous Graft; *CVC* Central Venous Catheter; *spKt/V* Single-pool Kt/V-urea; *URR* Urea reduction ratio

As shown in Table S1, before patients with DAP and AVF were matched, there were significant differences in gender (*P=*0.036) and CCI (*P*=0.040) between the two groups. After factors of “center”, “gender”, “CCI” were matched, the differences in patients’ characteristics between the two groups were not statistically significant (*P>*0.05). Finally, 38 cases with DAP and 38 cases with AVF were obtained. The differences of Kt/V (*P*=0.131) and URR (*P*=0.250) were not statistically significant.

Before patients with DAP and AVG were matched, there were significant differences in gender, age and blood pump flow speed between the two groups (*P*<0.05). Case-control matching methods were performed by “center”, “gender”, “age”, and the differences in patients’ characteristics between the two groups were not statistically significant(*P>*0.05). Finally, 22 cases with DAP and 22 cases with AVG were obtained. The differences of Kt/V (*P*=0.859) and URR (*P*=0.474) were not statistically significant. See Table S2 for the details.

As shown in Table S3, before patients with DAP and CVC were matched, there were significant differences in gender, primary disease and blood pump flow speed between the two groups (*P*<0.05). Case-control matching methods were performed by “center”, “gender”, and “primary disease”. Finally, 22 cases with DAP and 22 cases with catheter were obtained. The differences in patients characteristics between the two groups were not statistically significant(*P>*0.05). There was no significant difference in value of spKt/V(*P*=0.075) and URR between the two groups (*P*=0.468).

## Discussion

A well-functioning VA is the key to ensuring sufficient hemodialysis and to improving the prognosis of hemodialysis patients [2]. For various reasons such as complications, vascular exhaustion, technical and economic problems, some patients cannot choose arteriovenous access or catheters as VA for hemodialysis. DAP has the advantages of being used for emergency dialysis and low cost. One more choice means one more chance to live for hemodialysis patients.

Our results showed that there were 38(0.43%) patients received maintenance hemodialysis with DAP in 30 hemodialysis centers, Which was less than 1.8% of superficialization of artery reported by the Statistical Survey Committee of the Japanese Society for Dialysis Therapy among 172,244 patients surveyed [14]. The median duration of DAP was 1428 days with a interquartile range (141~ 2950 days), which not only solved the needs of temporary hemodialysis, but also met the requires of long-term hemodialysis in certain patients. When it comes to reasons for hemodialysis with DAP, 19 (50%) patients claimed to be unwilling to afford the cost of AVF creation or maintenance since DAP could maintain their hemodialysis treatment. All patients in this survey have already established vascular access before applying DAP for more than once, and even up to 7 times. AVFs are the preferred type of access, but the cost associated with creation and maintenance remains high [15]. The median annual overall cost for each hemodialysis patient was 87,125 Renminbi and more than one-third of the spending was related to VA maintenance [16]. In fact, both developing and developed countries bear the huge cost of VA maintenance [17]. Although the Chinese government is committed to having universal health coverage, as some medical insurance is voluntary, some patients fail to go through the procedures as required, which affects the reimbursement of medical expenses. In addition, the extra costs undoubtedly increase the burden on hemodialysis patients since their work ability was impaired [18]. Thus, patients would rather rely on DAP for treatment since the DAP could work successfully with little cost. Secondly, 39.5% of patients expressed the reason for DAP was that the matured arteriovenous fistula cannot be created because of the poor condition of vascular or cardiac function. The rapid growth of the aging population and the high prevalence of comorbidities, particularly diabetes mellitus and peripheral vascular disease, in patients requiring hemodialysis inevitably deteriorate the ability to construct and maintain a conventional AVF because of these patients’ insufficient vascular adaptability. In addition, an arteriovenous shunt can increase the heart burden. A decrease in systemic vascular resistance may produce cardiac symptoms which can lead to heart failure due to the arteriovenous shunt [19]. Such patients virtually face death in the absence of hemodialysis therapy, as a renal transplant is not readily available especially requiring emergency hemodialysis. CVC is effective but related to high infection complications impacting the image, comfort and even life of the patients. The KDOQI clinical practice guideline encouraged the selection of appropriate vascular access according to patient's ESKD Life-Plan [4] DAP is sure to be a good choice in a certain situation. However, DAP was limited due to complications such as hemorrhage, infection, vascular insufficiency, ischemia, thrombosis, embolization, and neuronal or adjacent structure injury [20]. Therefore, we must evaluate necessity and feasibility of DAP for patients undergoing hemodialysis.

In this study, 24 cases (63.2%) of DAP were on the radial artery. Radial artery, being easily accessible because of its superficial location, is one of the most preferred sites for DAP and has a low rate of procedural complications [9]. Radial artery puncture is a relatively safe procedure with an incidence of permanent ischemic complications of 0.09% [21]. Apart from cannulation on peripheral autologous arteries, 6 cases (15.8%) were punctured on anastomotic sites of abandoned fistula, which is so full and superficial as to puncture easily. It can be seen that choosing the correct cannulation site is particularly important to reduce complications. In addition, the puncture technique of nurse is vital to the success of DAP. In this survey, all DAP procedures were conducted by senior nurses. As reported, improving the puncture technique of nursing staff and using Doppler ultrasound guided puncture technique can improve the success rate of puncture, avoid injury of blood vessels, and prevent complications such as aneurysm, hematoma and massive bleeding [22]. The time-to-hemostasis compression after needle withdrawal was 0.5~1 hour among 16(42.1%) patients, Which was slightly longer than AVF cannulation. The methods of hemostasis depend on the different puncture sites. The brachial artery is deeper compared to radial artery, so the time of manual compression should be longer. Lower extremity with DAP should avoid walking before hemostasis. Accurate compressing point and suitable time to hemostasis could effectively reduce the occurrence of hematoma, hemorrhage and pseudoaneurysm. A vascular closure device set onto the skin and punctured by dialysis needle prevents bleeding from the punctured vessels, making hand compression unnecessary [23].

When it comes to the pain of cannulation, 29 (76.3%) patients self-reported mild pain during cannulation (NRS: 0~3 scores), indicating that most patients can stand the pain caused by DAP. The feeling of pain might adversely affect patient compliance with dialysis and quality of life. For the patients with pain intolerance, injection of local anesthetic and music therapy could decrease the pain of DAP [24].

As for the complications of DAP, aneurysm or pseudoaneurysm was reported in 16(42.1%) patients, which was one of the most common complications of DAP [25]. A study involving 28 patients was reported that,1 patient suffer from an infected pseudoaneurysm formation associated with DAP and 2 patients required an aneurysmectomy during 3 years [11]. Another study was reported that patients with hypertension, atrial fibrillation, or chronic kidney disease were more likely to develop a pseudoaneurysm than those without these conditions [26]. Therefore, when facing with patients prone to develop pseudoaneurysm, nurses should be more cautious and find ways to free the patients from pseudoaneurysm. Thrombin and external compression may be effective in treating upper extremity pseudoaneurysms [27]. Another complication more often reported was infiltration 12(31.6%) in this study. Whether in arteriovenous access cannulation or DAP, infiltration is a very common complication [28]. Missed cannulation was a vital reason for infiltration [29]. Before cannulation, the risk of missed cannulation could be minimized by fully evaluating the characteristics of the patient and the qualified nurses [29]. Ultrasound-guided cannulation and choosing appropriate plastic cannulae could decrease complications such as needle injuries caused by needle displacement due to arterial pulsation and restlessness of patients [4].

The results of this study show that DAP can provide average blood pump speed of 224.21±26.57ml/min, which was similar to studies on superficial brachial artery [11], higher than direct vena puncturing on cephalic vein as the inflow to dialyzer with a blood roller pump at a low rate of 120 to150ml/min [30], Moreover, the results of this study show that DAP was comparable to dialysis adequacy of other VA types. And compared with patients using CVC, there seems to be higher dialysis adequacy. It may be that when the VA is CVC, repeated circulation occurs and the inadequacy of dialysis happens for the relatively close distance between the inflow and outflow holes of the catheter [31], Therefore, DAP can achieve the required prescribed blood pump flow and adequacy of hemodialysis.

### Limitations

This research has inherent shortcomings in cross-sectional research. First, the patency of DAP was not included in this study. Second, the effect of DAP is affected by the nurse's puncture technique. Third, the key of successful cannulation of DAP depends on the patients themselves naturally superficial artery, which may be the selection bias we could not avoid. In addition, DAP are limitted with the complications of aneurysm or pseudoaneurysm. We suggest improving the puncture technique and the safety of DAP with Color Doppler ultrasound or other advanced equipment and patients education rather than abandoning the direct arterial puncture technique that may paly an important role in saving patients under emergency conditions.

## Conclusions

In conclusion, DAP can meet the needs of prescription hemodialysis. Yet it may be limited for its difficulty of puncture and complications such as aneurysm or pseudoaneurysm, it is necessary for well-qualified nurses to fully evaluate the characteristics of patients, carefully puncture and provide the knowledge of avoiding complications to patients. We found that with the equipment and well evaluation to ensure successful DAP, this shuntless access permits adequate blood flow and has theoretical advantages for some patients, particularly those with impaired cardiac function. Although the patients in our study were long-term dependent on DAP for hemodialysis with various reasons, we do not recommend DAP as a long-term vascular access if better options are available. DAP provides immediate availability for hemodialysis, so it may be an appropriate choice saving patients in emergent or compelling situation. Consequently, we call for this DAP technology not to be lost.

## Supplementary Information

**Additional file 1: Table S1.** Demographics characteristics of participants with DAP and AVF. **Table S2.** Demographics characteristics of participants with DAP and AVG. **Table S3.** Demographics characteristics of participants with DAP and CVC.

## Data Availability

The datasets generated during and analyzed during the current study are not publicly available due to individual privacy restriction on medical records but are available from the corresponding author on reasonable request.

## Abbreviations

MHD: Maintenance hemodialysis
VA: Vascular access
AVF: Arteriovenous fistula
AVG: Arteriovenous graft
CVC: Central venous catheter
DAP: Direct arterial puncture
NRS: Numeric Rating Scales
URR: Urea reduction ratio
SD: Standard deviation
IQR: Interquartile range
CCI: Charlson Comorbidity Index
