# Supplementary material for: Direct arterial puncture for hemodialysis, a neglected but simple and valuable vascular access
Source: BMC Nephrol. 2022 Jun 23;23:221. doi: 10.1186/s12882-022-02836-1 (PMC9219229; doi:10.1186/s12882-022-02836-1)
Supplement: Supplementary file 1 — Additional file 1: Table S1. Demographics characteristics of participants with DAP and AVF. Table S2. Demographics characteristics of participants with DAP and AVG. Table S3. Demographics characteristics of participants with DAP and CVC. [file 12882_2022_2836_MOESM1_ESM.doc]

| **Table S1** Demographics characteristics of participants with DAP and AVF | | | | | | | |
| --- | --- | --- | --- | --- | --- | --- | --- |
|  | **Before matching** | |  |  | **After matching** | |  |
| Items | DAP (n=38) | AVF (n=374) | *P* |  | DAP (n=38） | AVF (n=38) | *P* |
| Gender, n (%) |  |  | 0.036 |  |  |  | >0.999 |
| Male | 29(76.3) | 220(58.8) |  |  | 29（76.3） | 29(76.3) |  |
| Female | 9(23.7) | 154(41.2) |  |  | 9（23.7） | 9(23.7) |  |
| Age, year, mean±SD | 58.95±11.86 | 58.66±14.34 | 0.904 |  | 58.94±11.86 | 58.82±15.48 | 0.967 |
| BMI | 21.20±3.76 | 21.77±3.99 | 0.401 |  | 21.29±4.58 | 23.02±4.40 | 0.056 |
| Primary disease, n(%) |  |  | 0.212 |  |  |  | 0.792 |
| Chronic glomerulonephritis | 15(39.5) | 126(33.7) |  |  | 15(39.5) | 13(34.2) |  |
| Diabetic nephropathy | 8(21.1) | 99(26.5) |  |  | 8(21.1) | 8(21.1) |  |
| Hypertensive nephropathy | 5(13.2) | 70(18.7) |  |  | 5(13.2) | 9(23.7) |  |
| Obstructive nephropathy | 5(13.2) | 61(16.3) |  |  | 5(13.2) | 3(7.9) |  |
| Others | 5(13.2) | 18(4.8) |  |  | 5(13.2) | 5(13.2) |  |
| CCI，median (IQR) | 3(2,5) | 3(2,3) | 0.040 |  | 3(2,5) | 3(2,5) | 0.272 |
| Blood pump flow speed, ml/min, mean±SD | 224.21±26.57 | 217.83±19.09 | 0.157 |  | 224.21±26.57 | 217.11±19.58 | 0.189 |
| Hemodialysis time,hour,mean±SD | 3.83±0.54 | 3.91±0.32 | 0.368 |  | 3.83±0.54 | 3.96±0.18 | 0.737 |
| ALB, g/L, mean±SD | 35.96±4.42 | 36.86±2.80 | 0.228 |  | 35.96±4.42 | 36.54±2.69 | 0.494 |
| Hemoglobin, g/L, mean±SD | 108.24±15.75 | 108.12±12.39 | 0.069 |  | 108.24±15.75 | 108.79±12.05 | 0.864 |
| WBC,10^9/L, mean±SD | 5.94±2.18 | 6.16±2.31 | 0.580 |  | 5.94±2.18 | 6.21±2.54 | 0.620 |
| PLT,10^9/L, mean±SD | 171.66±68.48 | 175.99±49.11 | 0.706 |  | 171.66±68.48 | 182.17±54.07 | 0.460 |
| CRP, mg/L, median (IQR) | 5.3(1.00, 12.50) | 3.27(1.40,7.10) | 0.464 |  | 5.3(1.00,12.50) | 2.00(1.19,5.52) | 0.430 |
| Calcium, mmol/L, mean±SD | 2.26±0.18 | 2.30±0.20 | 0.344 |  | 2.26±0.18 | 2.24±0.14 | 0.562 |
| Potassium, μmol/L, mean±SD | 4.68±0.63 | 4.76±0.71 | 0.511 |  | 4.68±0.63 | 4.73±0.86 | 0.770 |
| Phosphate, μmol/L, mean±SD | 1.84±0.53 | 1.90±0.57 | 0.498 |  | 1.84±0.53 | 1.98±0.57 | 0.274 |
| iPTH, pg/ml, median (IQR) | 241.60(135.70,355.90) | 312.50(168.35,538.85) | 0.283 |  | 241.60(135.70,355.90) | 117.30(117.55,428.00) | 0.957 |
| UA, μmol/L, mean±SD | 440.22±112.15 | 462.93±99.74 | 0.187 |  | 440.22±112.15 | 444.52±80.47 | 0.848 |
| LDL,mmol/L, mean±SD | 2.80±1.43 | 2.63±0.87 | 0.484 |  | 2.80±1.43 | 2.64±0.92 | 0.572 |
| URR, mean±SD | 0.66±0.12 | 0.68±0.07 | 0.265 |  | 0.66±0.12 | 0.68±0.08 | 0.250 |
| KTV, mean±SD | 1.36±0.30 | 1.46±0.33 | 0.065 |  | 1.36±0.30 | 1.47±0.35 | 0.131 |
| DAP, direct arterial puncture; AVG, Arteriovenous Graft; SD, standard deviation; BMI, body mass index; IQR, interquartile range; CCI, Charlson Comorbidity Index; ALB, albumin Hemoglobin; WBC, white blood cell; PLT, platelet count; CRP, C-reactive protein; iPTH, intact parathyroid hormone; UA, uric acid; LDL,low-density lipoprotein; URR, Urea reduction ratio; spKt/V, single-pool Kt/V-urea | | | | | | | |
|

| **Table S2 Demographics characteristics of participants with DAP and AVG** | | | | | | | |
| --- | --- | --- | --- | --- | --- | --- | --- |
|  | **Before matching** | |  |  | **After matching** | |  |
| Items | DAP (n=38) | AVG (n=33) | *P* |  | DAP (n=22） | AVG (n=22) | *P* |
| Gender, n (%) |  |  | 0.015 |  |  |  | >0.999 |
| Male | 29（76.3） | 16(48.5) |  |  | 13(59.1) | 13(59.1) |  |
| Female | 9（23.7） | 17(51.5) |  |  | 9(40.9) | 9(40.9) |  |
| Age, year, mean±SD | 58.95±11.86 | 66.39±13.57 | 0.016 |  | 65.91±9.18 | 66.23±9.48 | 0.91 |
| BMI, mean±SD | 21.20±3.76 | 21.40±3.15 | 0.079 |  | 20.48±2.90 | 21.68±1.83 | 0.122 |
| Primary disease, n(%) |  |  | 0.055 |  |  |  | 0.386 |
| Chronic glomerulonephritis | 15(39.5) | 7(21.2) |  |  | 8(36.4) | 4(18.2) |  |
| Diabetic nephropathy | 8(21.1) | 11(33.3) |  |  | 6(27.3) | 8(36.4) |  |
| Hypertensive nephropathy | 5(13.2) | 7(21.2) |  |  | 3(13.6) | 5(22.7) |  |
| Obstructive nephropathy | 5(13.2) | 8(24.2) |  |  | 3(13.6) | 5(22.7) |  |
| Others | 5(13.2) | 0(0.0) |  |  | 2(9.1) | 0(0.0) |  |
| CCI，median (IQR) | 3(2,5) | 3(2,4) | 0.865 |  | 3(2,5) | 3(2,4) | 0.896 |
| Blood pump flow speed, ml/min, mean±SD | 224.21±26.57 | 212.12±15.36 | 0.020 |  | 219.55±22.78 | 213.64±14.65 | 0.313 |
| Hemodialysis time,hour,mean±SD | 3.83±0.54 | 3.86±0.30 | 0.809 |  | 3.89±0.41 | 3.85±0.30 | 0.737 |
| ALB, g/L, mean±SD | 35.96±4.42 | 36.37±2.18 | 0.616 |  | 36.30±3.09 | 36.43±2.42 | 0.870 |
| Hemoglobin, g/L, mean±SD | 99.92±26.73 | 107.27±10.86 | 0.126 |  | 111.09±16.44 | 108.14±11.17 | 0.489 |
| WBC,10^9/L, mean±SD | 5.94±2.18 | 6.48±2.31 | 0.312 |  | 5.99±2.32 | 6.21±2.16 | 0.742 |
| PLT,10^9/L, mean±SD | 171.66±68.48 | 173.06±55.07 | 0.925 |  | 176.82±77.63 | 177.68±61.59 | 0.968 |
| CRP, mg/L, median (IQR) | 5.3(1.00, 12.50) | 2.66(0.88,8.22) | 0.403 |  | 6.00(2.345,12.750) | 2.56(0.94,8.350) | 0.089 |
| Calcium, mmol/L, mean±SD | 2.26±0.18 | 2.30±0.16 | 0.335 |  | 2.27±0.19 | 2.30±0.16 | 0.635 |
| Potassium, μmol/L, mean±SD | 4.68±0.63 | 4.81±0.64 | 0.406 |  | 4.80±0.69 | 4.82±0.71 | 0.920 |
| Phosphate, μmol/L, mean±SD | 1.84±0.53 | 1.82±0.45 | 0.920 |  | 1.73±0.47 | 1.80±0.45 | 0.600 |
| iPTH, pg/ml, median (IQR) | 241.60(135.70, 355.90) | 335.5(198.80,618.10) | 0.161 |  | 241.60(126.48,658.45) | 378.90(233.93,599.85) | 0.209 |
| UA, μmol/L, mean±SD | 440.22±112.15 | 437.47±78.36 | 0.907 |  | 455.96±125.36 | 432.51±75.71 | 0.465 |
| LDL,mmol/L, mean±SD | 2.80±1.43 | 2.40±0.76 | 0.142 |  | 2.93±1.56 | 2.47±0.75 | 0.460 |
| URR, mean±SD | 0.66±0.12 | 0.69±0.07 | 0.111 |  | 0.67±0.09 | 0.69±0.07 | 0.474 |
| KTV, mean±SD | 1.36±0.30 | 1.44±0.41 | 0.369 |  | 1.41±0.29 | 1.43±0.42 | 0.859 |
| DAP, direct arterial puncture; AVG, Arteriovenous Graft; SD, standard deviation; BMI, body mass index; IQR, interquartile range; CCI, Charlson Comorbidity Index; ALB, albumin Hemoglobin; WBC, white blood cell; PLT, platelet count; CRP, C-reactive protein; iPTH, intact parathyroid hormone; UA, uric acid; LDL,low-density lipoprotein; URR, Urea reduction ratio; spKt/V, single-pool Kt/V-urea | | | | | | | |
|

| **Table S3 Demographics characteristics of participants with DAP and CVC** | | | | | | | |
| --- | --- | --- | --- | --- | --- | --- | --- |
|  | **Before matching** | |  |  | **After matching** | |  |
| Items | DAP (n=38) | CVC (n=81) | *P* |  | DAP (n=22） | CVC (n=22) | *P* |
| Gender, n (%) |  |  | 0.002 |  |  |  | >0.999 |
| Male | 29（76.3） | 37(45.7) |  |  | 14(63.6) | 14(63.6) |  |
| Female | 9（23.7） | 44(54.3) |  |  | 8(36.4) | 8(36.4) |  |
| Age, year, mean±SD | 58.95±11.86 | 62.44±17.25 | 0.261 |  | 62.14±12.19 | 60.14±16.31 | 0.64 |
| BMI, mean±SD | 21.20±3.76 | 20.83±2.95 | 0.384 |  | 21.29±4.58 | 19.58±3.08 | 0.095 |
| Primary disease, n(%) |  |  | ＜0.001 |  |  |  | >0.999 |
| Chronic glomerulonephritis | 15(39.5) | 8(9.9) |  |  | 4(18.2) | 4(18.2) |  |
| Diabetic nephropathy | 8(21.1) | 43(53.1) |  |  | 7(31.8) | 7(31.8) |  |
| Hypertensive nephropathy | 5(13.2) | 12(14.8) |  |  | 2(9.1) | 2(9.1) |  |
| Obstructive nephropathy | 5(13.2) | 8(9.9) |  |  | 4(18.2) | 4(18.2) |  |
| Others | 5(13.2) | 10(12.3) |  |  | 5(22.7) | 5(22.7) |  |
| CCI，median (IQR) | 3(2,5) | 3(2,4) | 0.538 |  | 3(2,5) | 3(2,5) | 0.757 |
| Blood pump flow speed, ml/min, mean±SD | 224.21±26.57 | 200.25±10.24 | <0.001 |  | 209.55±16.76 | 202.27±14.78 | 0.134 |
| Hemodialysis time,hour,mean±SD | 3.83±0.54 | 3.72±0.42 | 0.212 |  | 3.71±0.68 | 3.75±0.40 | 0.789 |
| ALB, g/L, mean±SD | 35.96±4.42 | 35.83±2.71 | 0.869 |  | 35.71±4.47 | 35.37±2.89 | 0.767 |
| Hemoglobin, g/L, mean±SD | 99.92±26.73 | 108.06±12.33 | 0.080 |  | 106.91±13.83 | 107.41±15.18 | 0.910 |
| WBC,10^9/L, mean±SD | 5.94±2.18 | 6.19±2.70 | 0.619 |  | 5.73±2.35 | 5.59±1.88 | 0.833 |
| PLT,10^9/L, mean±SD | 171.66±68.48 | 175.23±42.69 | 0.769 |  | 168.86±73.17 | 166.86±30.72 | 0.907 |
| CRP, mg/L, median (IQR) | 5.3(1.00, 12.50) | 5.90(2.00,8.00) | 0.513 |  | 6.48(2.35,12.23) | 5.70(2.00,8.29) | 0.319 |
| Calcium, mmol/L, mean±SD | 2.26±0.18 | 2.27±0.19 | 0.875 |  | 2.25±0.17 | 2.22±0.21 | 0.521 |
| Potassium, μmol/L, mean±SD | 4.68±0.63 | 4.66±0.69 | 0.878 |  | 4.67±0.64 | 4.62±0.69 | 0.826 |
| Phosphate, μmol/L, mean±SD | 1.84±0.53 | 2.00±0.57 | 0.135 |  | 1.79±0.60 | 2.06±0.56 | 0.135 |
| iPTH, pg/ml, median (IQR) | 241.60(135.70, 355.90) | 286.50(141.10,426.50) | 0.820 |  | 232.85(98.46,488.60) | 311.00(155.10,727.28) | 0.348 |
| UA, μmol/L, mean±SD | 440.22±112.15 | 448.85±123.20 | 0.423 |  | 453.07±122.73 | 453.65±91.50 | 0.986 |
| LDL,mmol/L, mean±SD | 2.80±1.43 | 2.52±0.87 | 0.275 |  | 2.84±1.32 | 2.12±0.74 | 0.030 |
| URR, mean±SD | 0.66±0.12 | 0.69±0.06 | 0.150 |  | 0.67±0.13 | 0.69±0.06 | 0.468 |
| KTV, mean±SD | 1.35±0.27 | 1.21±0.41 | 0.022 |  | 1.41±0.28 | 1.24±0.37 | 0.075 |
| DAP, direct arterial puncture; CVC, Central Venous Catheter; SD, standard deviation; BMI, body mass index; IQR, interquartile range; CCI, Charlson Comorbidity Index; ALB, albumin Hemoglobin; WBC, white blood cell; PLT, platelet count; CRP, C-reactive protein; iPTH, intact parathyroid hormone; UA, uric acid; LDL,low-density lipoprotein; URR, Urea reduction ratio; spKt/V, single-pool Kt/V-urea | | | | | | | |
|
